# 铜介导的磁性表面分子印迹聚合物用于棉仁中棉酚的高效特异性分离

**DOI:** 10.3724/SP.J.1123.2024.12006

**Published:** 2026-01-08

**Authors:** Shuling YANG, Yu CAO, Kunlin HE, Shun FENG, Chungu ZHANG, Lianhai SHAN

**Affiliations:** 西南交通大学生命科学与工程学院，四川 成都 610031; School of Life Science and Engineering，Southwest Jiaotong University，Chengdu 610031，China

**Keywords:** 棉酚, 铜介导, 分子印迹聚合物, 磁分离, 棉仁, gossypol （GOS）, copper mediated, molecularly imprinted polymer （MIP）, magnetic separation, cotton kernels

## Abstract

棉花是中国重要的经济作物，其棉籽是重要的油料和蛋白质资源。但棉籽中含有的天然活性产物棉酚（gossypol，GOS）对哺乳动物具有生育毒性和生长抑制作用，这严重限制了棉籽资源的综合利用。本研究创新性地设计了一种基于金属配位机制的磁性表面分子印迹聚合物（GOS/MIP），通过自由基聚合法将铜离子介导的印迹位点精准锚定于功能化磁核的表面，实现棉酚的高效特异性分离与资源化增值的协同目标。表征结果证实，GOS/MIP是粒径为400~500 nm的核壳结构球形颗粒，具有优异的磁响应性（47.78 emu/g），可在7 s内实现快速磁分离。其具有优异的与GOS结合的能力，120 min内的最大吸附容量为74.01 mg/g，印迹因子（IF）达6.48，此外，GOS/MIP在复杂基质中对GOS表现出高选择性和高特异性，且具有良好的稳定性和重复使用性。以GOS/MIP为分散固相萃取吸附剂与高效液相色谱结合，所建立的方法在5~200 μg/mL的GOS质量浓度范围内具有良好的线性关系（*R* ^2^＞0.999），检出限为0.024 μg/mL，加标回收率为95.1%~98.7%，相对标准偏差≤2.4%。进一步模拟工业化的GOS分离场景，仅消耗50 mL溶剂和50 mg GOS/MIP即可从10 g棉仁样品中分离得到3 mg GOS，回收率达77.0%~83.3%。这项工作有效克服了传统GOS分离过程中选择性差、环境负担大的瓶颈，为天然产物的高值化利用提供了绿色、高效的新策略，兼具基础研究创新性与产业化应用潜力。

## Abstract (en)

Cotton is an economically important crop in China. Cotton fibers are widely used in the textile industry and cottonseed is a key source of edible oils and proteins. Cottonseed contains gossypol （GOS）， a natural bioactive compound that exhibits medicinal properties， including antitumor and antiviral activities； it is also a potential contraceptive agent. Despite these benefits， GOS has some critical drawbacks， as it is associated with side effects such as antifertility and mammalian growth inhibition that limit the comprehensive utilization of cottonseed resources. Consequently， GOS needs to be efficiently and specifically separated for safe resource utilization. Conventional molecularly imprinted polymers （MIPs） have limitations as they often bind to targets in a manner that is too rigid or fragile， which leads to significantly lower specificities and mass-transfer rates. Accordingly， a novel copper-ion-mediated magnetic surface molecularly imprinted polymer （GOS/MIP） was designed and prepared to solve these problems. GOS/MIP integrates three key strategies： metal coordination， surface imprinting， and magnetic separation. Copper（Ⅱ） ions were introduced as functional “bridges” between the functional monomer and template， which resulted in softer and more flexible interactions and more homogeneous imprinted cavities. The imprinting sites were anchored to the surfaces of the functionalized magnetic cores， which accelerated the adsorption and desorption processes and simplified the preparation and separation steps. Free-radical polymerization enabled precise synthesis. GOS/MIP was thoroughly characterized， with scanning electron microscopy （SEM） and transmission electron microscopy （TEM） used to observe its morphology， and a vibrating sample magnetometer （VSM） used to measure magnetic properties. Energy-dispersive spectroscopy （EDS） was used to analyze the elemental composition， with Fourier-transform infrared （FT-IR） spectroscopy used to examine chemical bonding. The results confirmed the existence of a core-shell structure and that the imprinted layer had been successfully prepared. GOS/MIP was found to be composed of spherical particles 400–500 nm in diameter. The material also exhibited an excellent magnetic response， with a saturation magnetization of 47.78 emu/g， which enabled magnetic separation within only 7 s， thereby ensuring rapid recovery under practical conditions. Performance studies revealed that GOS/MIP exhibits an excellent binding ability for GOS， with equilibrium achieved in 120 min. The apparent maximum adsorption capacity and imprinting factor （IF） were determined to be 74.01 mg/g and 6.48， respectively. Selective and competitive binding experiments showed that GOS/MIP is highly specific for GOS in complex matrices， and pH binding experiments revealed that optimal binding occurs at pH 2–6， which covers most industrial processing conditions and highlights the large-scale industrial applications potential of the material. GOS/MIP retained its good adsorption capacity even after seven adsorption-desorption cycles， thereby demonstrating excellent stability and reproducibility. GOS/MIP was used as a dispersive solid-phase extraction （dSPE） adsorbent in combination with high-performance liquid chromatography （HPLC）. The developed method exhibited linearity in the 5–200 μg/mL range， with a high correlation coefficient （*R*²>0.999） and limits of detection and quantification （LOD and LOQ） of 0.024 and 0.079 µg/mL， respectively. Average recovery rates at three spiked concentrations （0.08， 0.24， and 0.80 μg/mL） ranged between 95.1% and 98.7%， with relative standard deviations （RSDs） below 2.4%. Only 50 mL of solvent and 50 mg of GOS/MIP were required in a simulated industrial GOS-separation process， which separated 3 mg of GOS from 10 g of cottonseed. GOS-recovery rates of 77.0%–83.3% were observed. The GOS/MIP-based separation method combines specificity， sustainability， and cost-effectiveness， thereby addressing the key challenges faced by traditional GOS-separation methods. In addition， it overcomes the toxicity limitations associated with the comprehensive utilization of cottonseed. Hence， cottonseed can be safely processed and cottonseed byproducts can be used in animal feed. This study provides a potent tool for the green， fast， and specific separation of high-purity products from plants； the basic research presented herein is both innovative and is potentially industrially applicable.

棉花是中国重要的经济作物，其棉纤维能制成丰富的纺织产品，棉籽则可作为食用油和蛋白质的重要来源^［1，2］^。然而，棉籽中天然存在的棉酚（gossypol，GOS，C_30_H_30_O_8_）是一种具有显著生物毒性的多酚羟基双萘醛类化合物，其会对哺乳动物产生抗生育^［3］^、生长抑制^［4］^等副作用。GOS的存在不利于棉籽油和棉籽粕的加工，严重制约棉籽的资源化利用。因此，开发高效、精准的GOS分离技术，可消除棉籽的毒性隐患，使其食用价值得到最大化。

目前GOS分离技术主要包括微生物发酵法^［5］^、溶剂萃取法^［6］^和固相萃取法^［7］^。其中，固相萃取法因操作条件温和、溶剂消耗少等优势备受关注，常用的吸附剂如氧化铝^［8］^、石墨化炭黑^［9］^等虽能实现部分分离，但普遍存在选择性差、传质效率低的问题。相较之下，分子印迹聚合物（molecularly imprinted polymers，MIPs）凭借更好的识别效率和选择性^［10-12］^，在从复杂基质中特异性分离目标分子方面展现出独特潜力^［13，14］^。传统MIPs通过共价键或非共价键与模板结合，但刚性作用力易导致印迹空腔分布不均^［15］^，而弱相互作用（如氢键）则存在稳定性差、溶剂敏感性高等缺点^［16］^，因此可能无法达到理想的GOS分离效果。

近年来，金属介导MIPs（metal-mediated MIPs）通过引入金属离子定向桥接功能单体和模板分子，实现了作用力“刚柔并济”的突破，有利于形成定向均匀的结合位点，构建更稳定、有效的印迹空腔，从而显著提高识别的特异性和选择性^［17，18］^。此外，表面分子印迹技术（surface molecularly imprinted technology）通过将印迹层包覆在固体基质表面，可以增加活性位点的可及性，加速吸附和解吸过程^［19，20］^。进一步使用Fe_3_O_4_纳米颗粒作为固体基质，可得到具有大比表面积和易接近结合位点的材料^［21］^，还可通过外部磁场大大简化和加快制备和分离过程^［22］^。上述策略可有效克服传统MIPs的固有缺点。

已有一些关于GOS的MIPs的报道。Zhao等^［23］^制备了具有双层结构的块体MIP，印迹因子（imprinting factor，IF）为3.85，实现了GOS的快速电化学检测，然而其依赖氢键作用，结合易受溶剂或其他物质的干扰。Zhi等^［24］^通过溶胶-凝胶法制备GOS的表面分子印迹聚合物，吸附容量高（204 mg/g）且平衡时间短（10 min），但IF值有限，为2.20。Zhi等^［25］^还通过自由基聚合法制备了GOS的MIPs，其吸附容量佳（632 mg/g），但仅比非印迹聚合物（NIPs）高13%~15%，且平衡时间较长（12 h），研磨过程使颗粒不规则，尺寸分布广。Wang等^［26］^通过本体聚合、溶胶-凝胶法和表面分子印迹法制备了3种GOS的MIPs并进行比较，本体聚合得到的MIP吸附容量高（564 mg/g），但相较于NIP（506.5 mg/g）没有明显优势，且其平衡时间较长（720 min），溶胶-凝胶表面印迹得到的MIP平衡时间短（40 min），吸附容量较大（120 mg/g），但IF值有限，为2.86。

本文采用金属介导-表面印迹-磁分离三元协同策略，设计并制备了一种新型Cu（Ⅱ）介导的磁性表面分子印迹材料GOS/MIP。GOS/MIP通过自由基聚合法制备得到，其以功能化Fe_3_O_4_为磁核，GOS为模板分子，丙烯酰胺（AM）为功能单体，乙二醇二甲基丙烯酸酯（EDGMA）为交联剂，Cu（Ⅱ）为连接功能单体和模板分子的“桥梁”。相较于现有报道，其充分整合了3种技术的优势，实现了协同优化结合作用力、印迹位点可及性与分离效率三大核心参数。通过多种方法对GOS/MIP进行了表征和性能测定，并成功应用于从植物样品棉仁中高效、绿色分离GOS的工业化模拟生产中。

## 1 实验部分

### 1.1 仪器、试剂与材料

Inspect-F50场发射扫描电子显微镜和G2F20高分辨透射电子显微镜（美国FEI公司），PPMS-9振动样品磁力计（美国Quantum Design公司），Octane Super能量色散光谱仪（美国Octane公司），XFlash 6130能谱仪（美国BRUKER公司），FTIR-650傅里叶变换红外光谱仪（天津港东科技发展股份有限公司），1525高效液相色谱仪（美国Waters公司），UV-1800PC紫外可见分光光度计（上海AOE仪器有限公司），EKUP-Ⅱ-20E纯水系统（四川宜科纯水设备有限公司），Nano-Zen 3600激光粒度仪（英国Malvern公司）。

AM（≥98%）、偶氮二异丁腈（AIBN，≥98%）、五水硫酸铜（Cu（SO_4_）_2_⋅5H_2_O，≥99%）、七水硫酸锌（Zn（SO_4_）_2_⋅7H_2_O，≥99.5%）、原硅酸四乙酯（TEOS，99%）、甲基丙烯酰氧基丙基三甲氧基硅烷（MPS，98%）、聚乙二醇4000（PEG-4000，生物超纯级）、醋酸钠（NaAC，98%）、阿霉素（DOX，98%）、多巴胺（DOP，≥99%）、土霉素（OTC，≥98%）、2-羟基-1-萘甲醛（HNA，98%）、2，6-二羟基萘-1，5-二甲醛（DHNDA，98%）、甲基丙烯酸（MAA，99%）、衣康酸（ITA，≥99%）、4-乙烯基吡啶（4-VP，99%），氨水（NH_3_⋅H_2_O，生物超纯级）、乙二醇（≥99.5%）、冰乙酸（≥99.5%）、氢氧化钠（≥98%）、甲酸（HPLC≥98%）均购自上海阿达玛斯试剂有限公司；EDGMA（98%）、诺氟沙星（NFX，≥98%）均购自阿拉丁试剂（上海）有限公司；六水合氯化铁（FeCl_3_⋅6H_2_O，99%）购自上海麦克林生化科技有限公司；GOS（≥98%）购自成都健腾生物有限公司；芦丁（RUT，≥98%）购自成都德思特生物技术有限公司；分析纯甲醇、乙醇、盐酸均购自成都市科隆化工试剂厂；色谱级乙腈和甲醇购自赛默飞世尔科技公司；实验用水为超纯水（18.2 MΩ·cm）。

### 1.2 材料制备

#### 1.2.1 Fe_3_O_4_制备

称取2.70 g FeCl_3_·6H_2_O溶解于20 mL乙二醇中，记为A。称取7.2 g NaAc溶解于另外20 mL乙二醇中，记为B。称取2 g PEG-4000溶于另外20 mL乙二醇中，记为C。将A、B、C混合并快速搅拌30 min后，转移至100 mL反应釜中，200 ℃下反应8 h。反应结束后将产物冷却至室温，通过外部磁场收集产物，用无水乙醇和去离子水洗涤数次后真空冷冻干燥12 h，得到Fe_3_O_4_保存备用。

#### 1.2.2 Fe_3_O_4_@SiO_2_制备

在Fe_3_O_4_纳米颗粒表面包覆二氧化硅层。称取1 g Fe_3_O_4_分散到100 mL无水乙醇-水混合溶液（4∶1，体积比），在31.4 rad/s机械搅拌下加入3 mL NH_3_·H_2_O和2 mL TEOS，室温反应12 h后，通过外部磁场分离产物，用无水乙醇和去离子水洗涤数次，真空冷冻干燥12 h，得到Fe_3_O_4_@SiO_2_保存备用。

#### 1.2.3 Fe_3_O_4_@SiO_2_@MPS制备

称取200 mg Fe_3_O_4_@SiO_2_分散于100 mL 10%冰醋酸中，在31.4 rad/s机械搅拌下加入1 mL MPS，60 ℃下反应5 h，反应结束待产物冷却至室温后，通过外部磁场分离产物，用无水乙醇和去离子水洗涤数次，真空冷冻干燥12 h，得到Fe_3_O_4_@SiO_2_@MPS保存备用。

#### 1.2.4 GOS/MIP制备

取13 mg GOS溶解于16 mL甲醇，取100 mg Cu（SO_4_）_2_⋅5H_2_O溶解于4 mL去离子水，将二者混匀，30 min后加入14 mg AM进行预组装，30 min后加入100 mg Fe_3_O_4_@SiO_2_@MPS，混匀1 h后加入190 µL EGDMA和16 mg AIBN，通氮除氧后，在31.4 rad/s机械搅拌、60 ℃下反应12 h。反应结束待产物冷却至室温后，磁分离产物，用甲醇-乙酸混合溶液（8∶2，体积比）洗脱数次以去除模板，然后用无水乙醇和去离子水将产物洗至中性，再加入0.2 mol/L Cu（Ⅱ）孵育30 min，孵育完成后用去离子水洗涤产物3次，真空冷冻干燥12 h，得到GOS/MIP保存备用。

NIP的制备过程除不加GOS外，其余步骤均相同。

### 1.3 吸附性能测试

#### 1.3.1 等温吸附实验

取4 mg GOS/MIP或NIP，用甲醇活化后分散到4 mL质量浓度为0.5~500 µg/mL的GOS标准溶液中。室温下旋转孵育2 h后，磁分离材料，用紫外可见分光光度计测定吸附前、后上清液在373 nm的吸光度，结合标准曲线法计算上清液中GOS的质量浓度，并利用公式（1）对GOS的吸附量进行计算，公式（2）对IF值进行计算。


$$Q=\frac{\left(C_{0}-C_{t}\right)\times V}{m}$$ （1）

$$IF=\frac{Q_{GOS/MIP}}{Q_{NIP}}$$ （2）

其中，*Q*（mg/g）表示材料的吸附量，*C*_0_（μg/mL）表示GOS溶液的初始质量浓度，*C_t_* （μg/mL）表示吸附后GOS的剩余质量浓度，*V*（mL）表示溶液的体积，*m*（mg）表示材料的质量；*Q*_GOS/MIP_和*Q*_NIP_（mg/g）分别表示GOS/MIP和NIP对GOS的吸附量。

#### 1.3.2 动力学吸附实验

取4 mg GOS/MIP或NIP，用甲醇活化后分散到4 mL 50 µg/mL的GOS标准溶液中，在室温下旋转孵育5、10、20、30、40、50、60、80、120、240 min后，磁分离材料，用紫外可见分光光度计测定吸附前、后上清液在373 nm的吸光度。

#### 1.3.3 选择性吸附实验

取4 mg GOS/MIP或NIP，用甲醇活化后分散到4 mL质量浓度均为50 μg/mL的GOS、RUT、OTC、DOX、DOP、NFX、DHNDA、HNA标准溶液中，室温下旋转孵育2 h后，磁分离材料，用紫外可见分光光度法测定并通过标准曲线计算上清液中GOS和各结构类似物的初始质量浓度和最终质量浓度。

#### 1.3.4 竞争性吸附实验

取4 mg GOS/MIP或NIP，用甲醇活化后分散到4 mL质量浓度各为50 μg/mL的GOS、RUT、OTC、DOX、DOP和NFX的混合溶液中，室温下旋转孵育2 h后，磁分离材料，用3 mL去离子水洗涤2次以去除非特异性吸附的组分，用3 mL甲醇-乙酸混合溶液（8∶2，体积比）洗涤2次以洗脱吸附的药物分子。分别收集洗涤液和洗脱液，真空冷冻干燥12 h后，用2 mL流动相复溶，通过 HPLC分析。

### 1.4 pH适用范围测试

取4 mg GOS/MIP或NIP，活化后分散到pH值在2~13范围内的GOS溶液中（用2% HCl水溶液和2% NaOH水溶液调节酸碱性）。室温下旋转孵育2 h后磁分离材料，通过紫外可见分光光度计测定吸附前、后上清液在373 nm的吸光度，并结合标准曲线计算GOS的质量浓度。

### 1.5 重复使用性测试

将4 mg GOS/MIP活化后分散到4 mL 50 µg/mL的GOS溶液中，室温下旋转孵育2 h后，将GOS/MIP自体系中磁分离并洗脱解吸，用Cu（Ⅱ）孵育再生后再次放入4 mL 50 µg/mL的GOS溶液中进行吸附。整个吸附-解吸-再生循环重复7次。测定和计算每次吸附前、后GOS的质量浓度。

### 1.6 方法评估与真实样本中GOS的分离

将10 g棉仁粗粉和100 mL丙酮-水（8∶2，体积比）混合溶液放入250 mL平底烧瓶中，在40 ℃下加热回流3 h，收集3次提取液经减压过滤、旋转蒸发得到0.7 g浸膏，复溶于50 mL甲醇中备用。取50 mg活化后的GOS/MIP分散于上述50 mL复溶液中，室温旋转孵育120 min后，磁分离GOS/MIP并收集上清液，材料用5 mL水洗涤2次，5 mL甲醇-乙酸（8∶2，体积比）混合溶液洗脱2次，合并洗脱液并真空干燥，通过HPLC分析处理前、后溶液中GOS的质量浓度。

### 1.7 分析条件

方法评估与真实样本中GOS的分离实验：色谱柱为Shimadzu WondaSil^® ^C18-WR柱（150 mm×4.6 mm， 5 µm），柱温为25℃；检测波长为280 nm，流动相为甲醇-水-甲酸（85∶15∶0.1，体积比），流速为1.0 mL/min，进样量为10 μL。

竞争性吸附实验：色谱柱、柱温、检测波长、流速和进样量与真实样本分离实验一致。流动相A和流动相B分别为0.1%甲酸水和0.1%甲酸甲醇。梯度洗脱程序：0~3.0 min，60%A；3.0~7.0 min，60%A~25%A；7.0~17.0 min，25%A~25%A；17.0~20.0 min，25%A~60%A。

## 2 结果与讨论

### 2.1 GOS/MIP的制备及条件优化

GOS/MIP的制备过程如图1所示。首先通过预组装步骤形成稳定的模板（GOS）-金属离子（Cu（Ⅱ））-单体（AM）三元复合物，这也是GOS/MIP成功制备的关键。然后以EDGMA为交联剂，AIBN为引发剂，通过自由基聚合法在磁性纳米颗粒表面包覆聚合物层，获得具有核壳结构的GOS/MIP，这种独特的形式有利于其与GOS的结合。

**图1:**
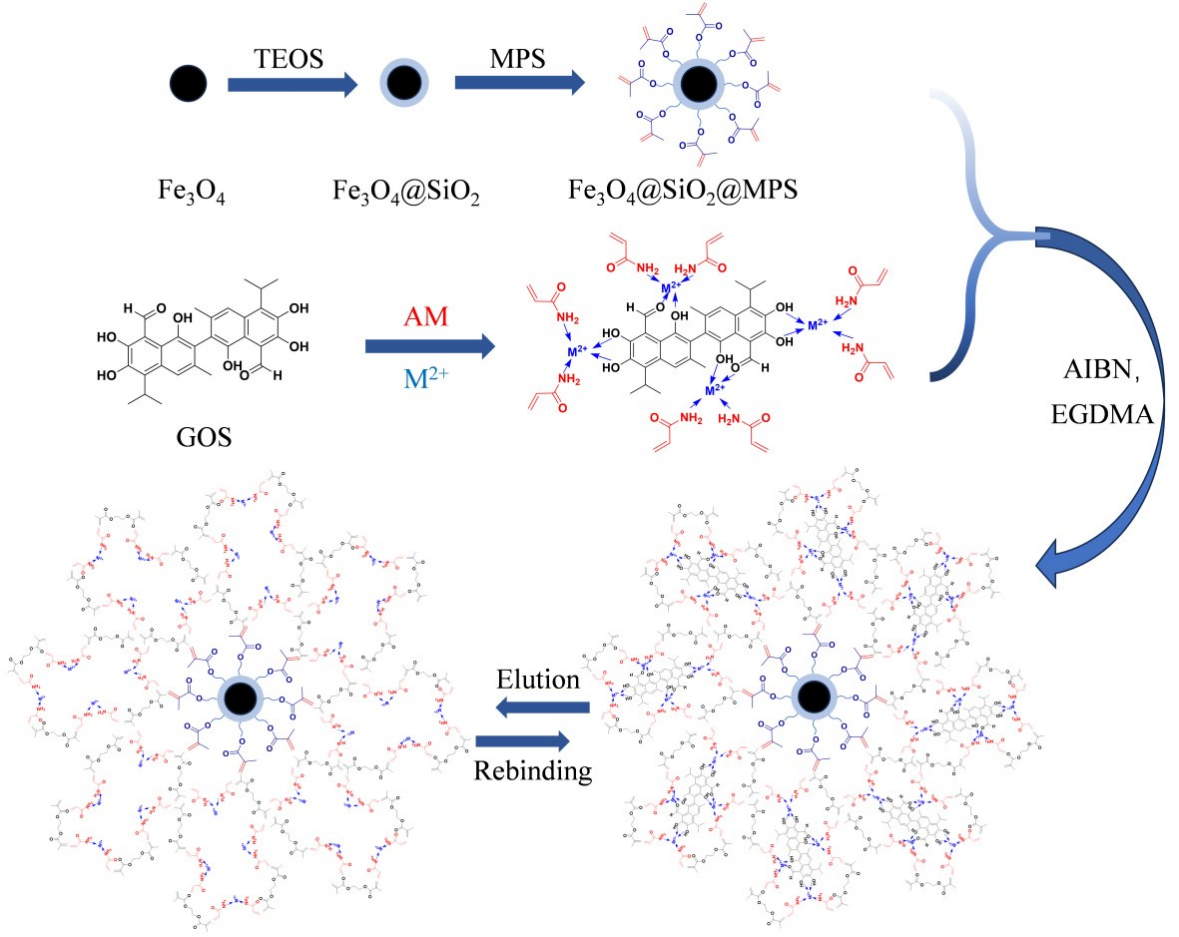
GOS/MIP的合成示意图

为了获得最佳的吸附能力，进行了优化实验。首先参考Nicholls等^［27］^的工作，将GOS与MAA、AM、4-VP、ITA（物质的量之比1∶4）在甲醇中振荡30 min，测定各溶液在190~500 nm的UV-Vis谱图以筛选功能单体；将GOS与Cu（Ⅱ）、Mn（Ⅱ）、Zn（Ⅱ）、Ni（Ⅱ）（物质的量之比1∶5）预混合30 min后加入单体二次振荡，测定各环节UV-Vis谱图。进一步通过单变量实验优化，按照GOS/MIP的条件和参数，制备4种单体不同的非金属介导MIP，以及制备4种金属离子不同的金属介导MIP进行考察。筛选结果如图2所示。

**图2:**
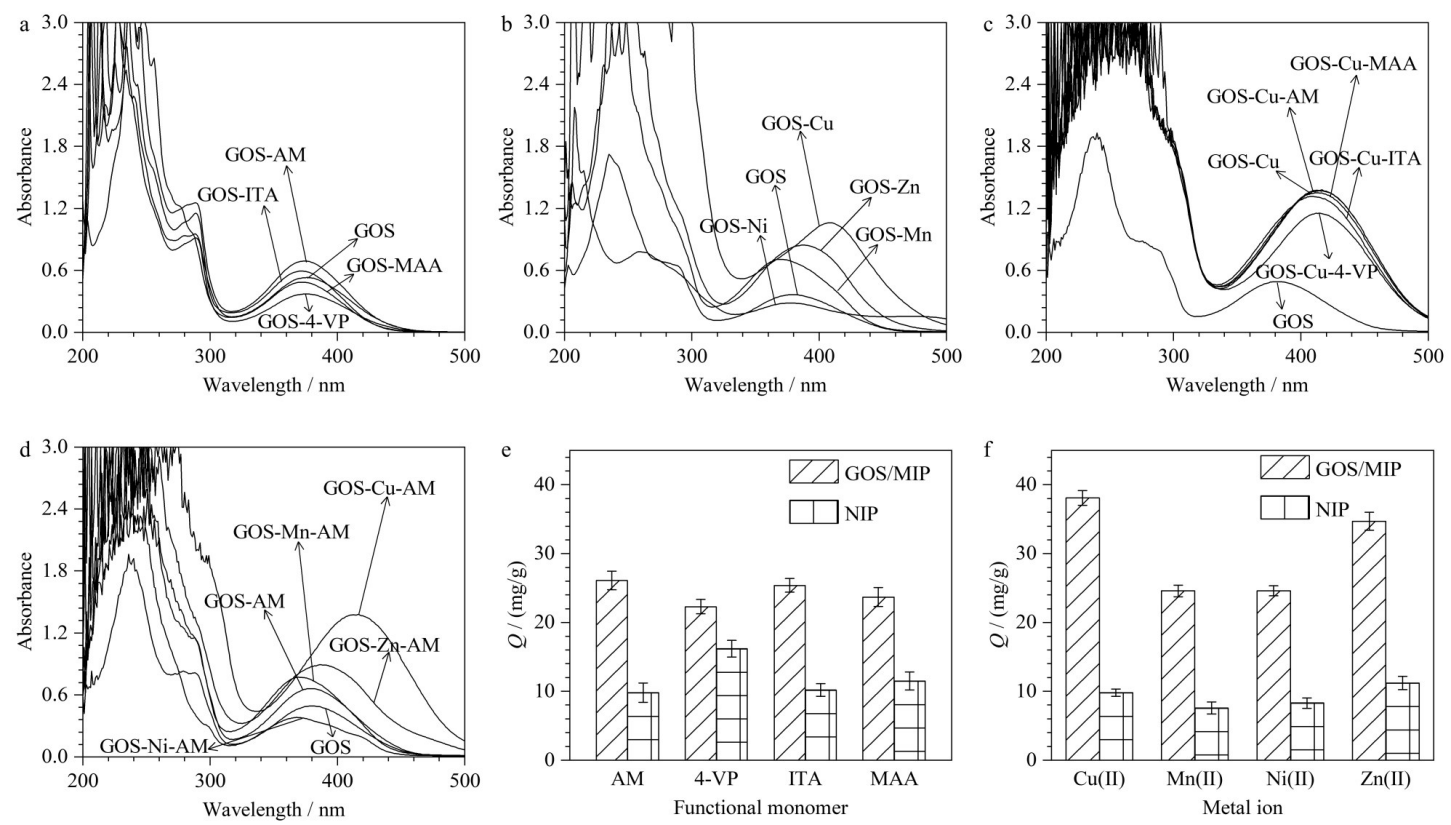
（a）GOS与不同功能单体、（b）GOS与不同金属离子、（c）GOS-Cu与不同功能单体及（d）GOS-AM与不同金属离子的紫外可见光谱图；（e）单体种类和（f）金属离子种类对GOS/MIP和NIP吸附GOS效果的影响（*n*=3）

由图2a和2b可知，功能单体AM和金属离子Cu（Ⅱ）使GOS的UV-Vis特征峰发生最显著的吸光度升高和红移效应；进一步分析发现，GOS-Cu（Ⅱ）-AM三元体系出现最强的吸收峰（图2c、2d）。这些结果表明GOS-Cu（Ⅱ）-AM是稳定的复合物。通过单变量实验验证发现，采用AM和Cu（Ⅱ）制备的MIP在吸附容量上均优于其他对应单体或金属离子的组合（图2e、2f）。

为进一步验证上述实验结果，采用密度泛函理论（DFT）进行量子化学计算（Gaussian 09软件，B3LYP-D3泛函结合色散校正，def2-svp基组）。首先对GOS与4种金属离子的复合物进行几何优化并计算各体系结合能（表1，图3），发现GOS与Cu（Ⅱ）结合时，其1号位结合能（Δ*E*_GOS，s1-M_=‒464.97 kJ/mol）和2号位结合能（Δ*E*_GOS，s2-M_=‒429.07 kJ/mol）的总和（Δ*E*_GOS-M_=Δ*E*_GOS，s1-M_+Δ*E*_GOS，s2-M_=‒894.04 kJ/mol）绝对值最大，表明GOS-Cu（Ⅱ）可通过金属离子与酚羟基氧、醛基氧的配位作用形成最稳定结构。进一步优化GOS-Cu（Ⅱ）与功能单体的配位体系（表2，图4），发现GOS-Cu（Ⅱ）与AM结合时，结合能（Δ*E*_GOS-M-monomer_=‒1 087.34 kJ/mol）绝对值最大，表明其能够形成能量最低的稳定配位构型。DFT计算结果与上述优化实验结果一致，最后选定Cu（Ⅱ）和AM用于GOS/MIP的合成。

**表1:** GOS与不同金属离子结合的结合能

Metal ion	Δ*E*_GOS，s1-M_/（kJ/mol）	Δ*E*_GOS，s2-M_/（kJ/mol）	Δ*E*_GOS-M_/（kJ/mol）
Cu（Ⅱ）	‒464.97	‒429.07	‒894.04
Mn（Ⅱ）	‒93.14	‒160.21	‒253.34
Ni（Ⅱ）	‒491.33	‒363.09	‒854.41
Zn（Ⅱ）	‒127.57	‒99.70	‒227.27

**图3:**
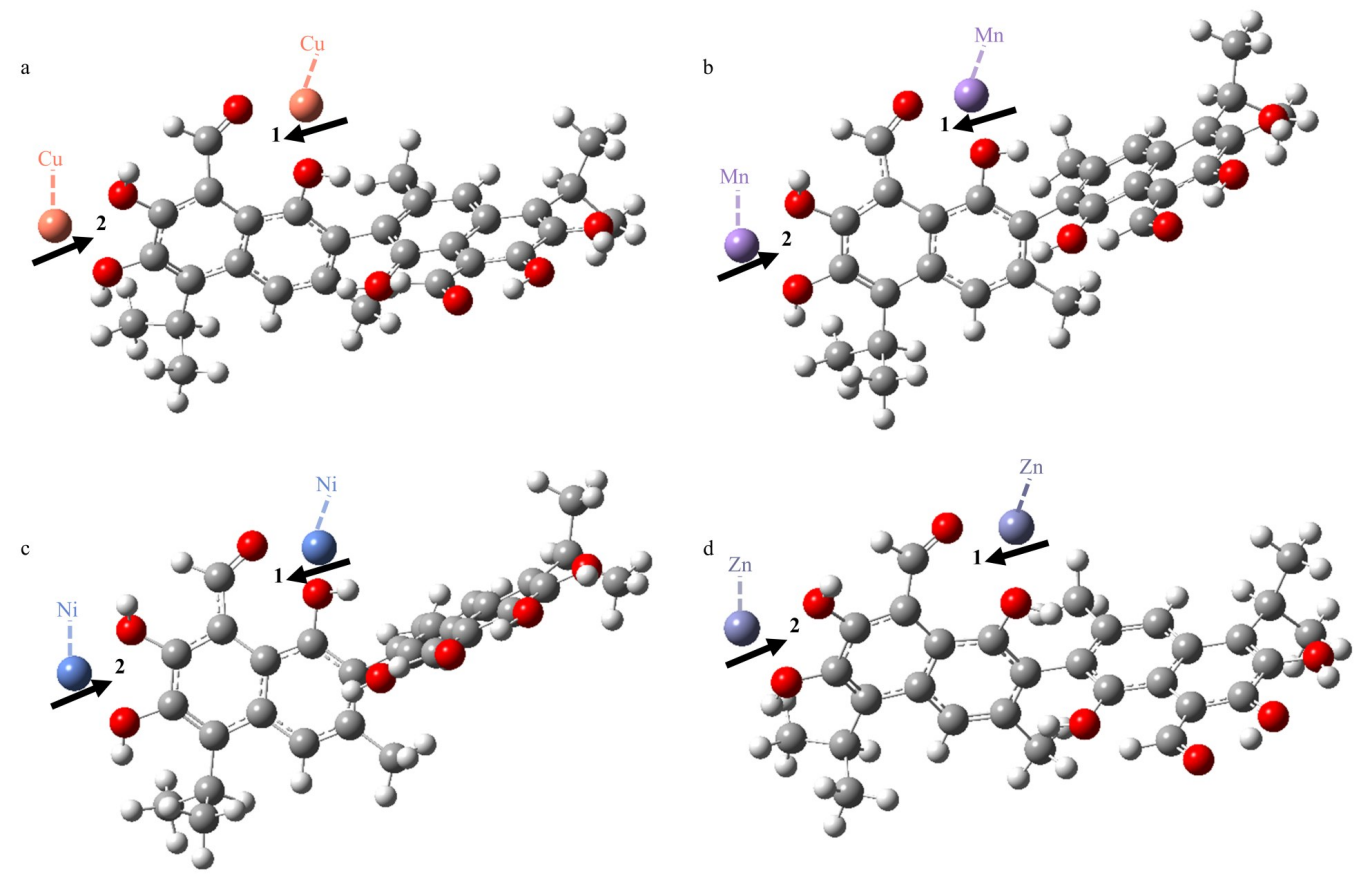
（a）GOS-Cu（Ⅱ）、（b）GOS-Mn（Ⅱ）、（c）GOS-Ni（Ⅱ）和（d）GOS-Zn（Ⅱ）的优化结构图

**表2:** GOS-Cu（Ⅱ）复合物与不同单体结合的结合能

Monomer	Δ*E*_GOS-M-monomer_/（kJ/mol）
AM	‒1087.34
4-VP	‒1054.12
ITA	‒446.85
MAA	‒643.58

**图4:**
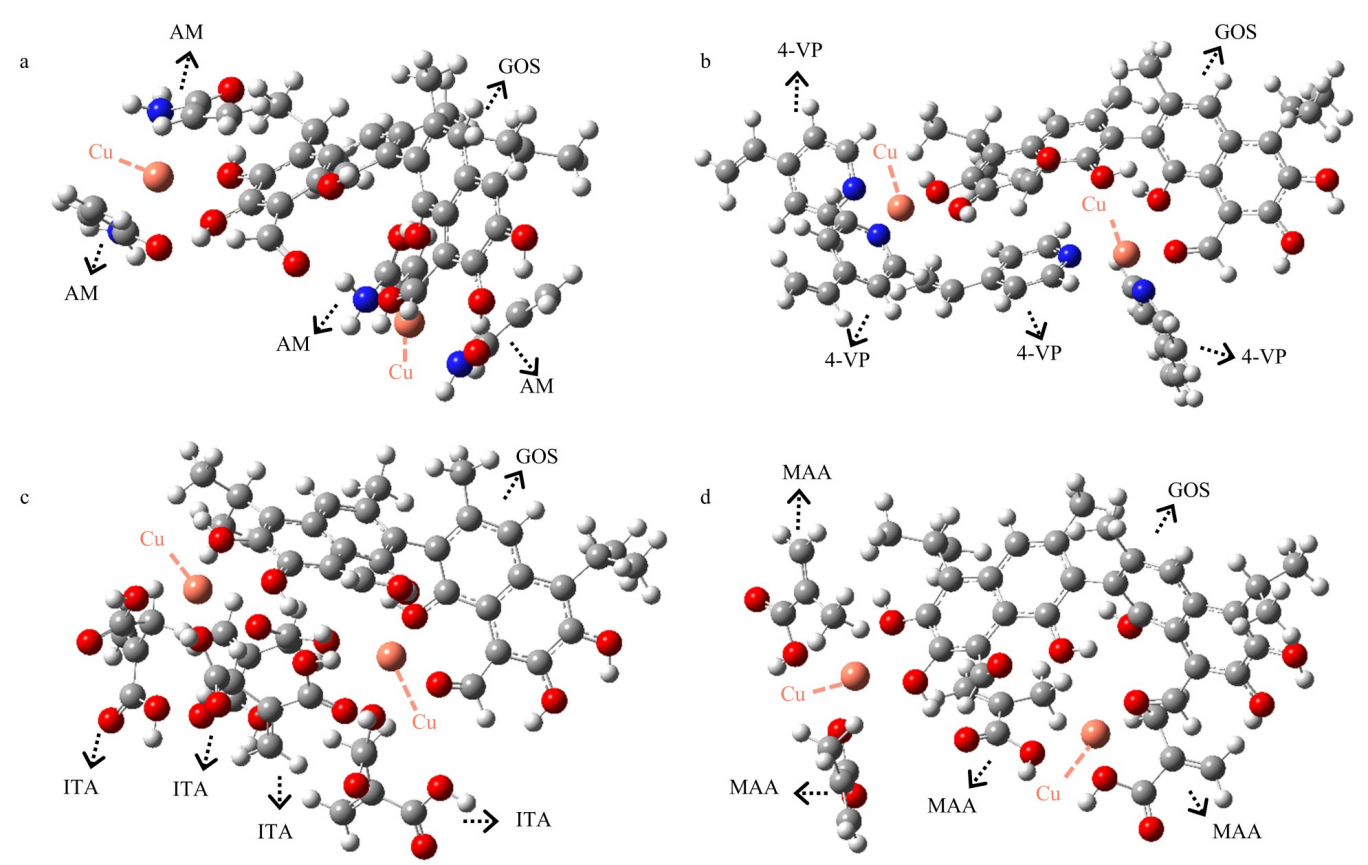
（a）GOS-Cu（Ⅱ）-AM、（b）GOS-Cu（Ⅱ）-4-VP、（c）GOS-Cu（Ⅱ）-ITA和（d）GOS-Cu（Ⅱ）-MAA的优化结构图

### 2.2 材料的表征

图5a为各材料的FT-IR图谱，3 441 cm^‒1^处的-OH特征峰和558 cm^‒1^处的Fe-O特征峰表明Fe_3_O_4_成功制备，并且在后续修饰、功能化和表面印迹过程中保持稳定。745、949和1 070 cm^‒1^处分别对应O-Si-O、Si-OH对称伸缩振动峰和Si-O不对称伸缩振动峰，表明SiO_2_层成功包覆。C=O（1 625 cm^‒1^）以及C=C（1 386 cm^‒1^）特征峰验证MPS功能化及印迹层键合成功。GOS/MIP和NIP由于成分相同呈现相似图谱，且均具有功能化磁核的所有特征峰。

**图5:**
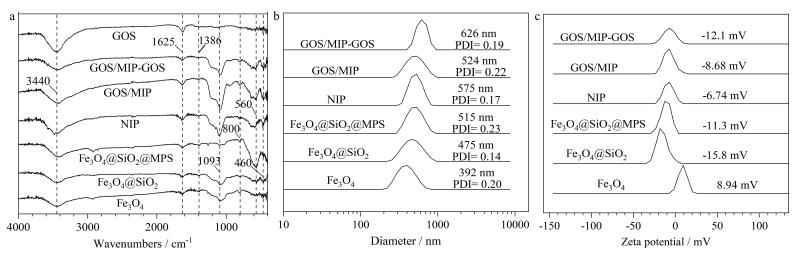
载体、NIP及模板吸附前后GOS/MIP的（a）红外光谱、（b）粒径分布和（c）Zeta电势 PDI： polymer dispersity index； GOS/MIP-GOS： GOS/MIP after adsorption of GOS.

粒径分布图（图5b）表明，Fe_3_O_4_的流体动力学粒径约为392 nm，经过层层包覆后纳米颗粒的粒径逐渐增大，表明GOS/MIP成功制备且能够吸附GOS。Zeta电位数据（图5c）显示，修饰SiO_2_、MPS、印迹层，以及吸附GOS后，材料的电位由正转负，进一步证明材料的成功制备，并且Fe_3_O_4_@SiO_2_和Fe_3_O_4_@SiO_2_@MPS相较于Fe_3_O_4_能更趋于稳定地悬浮于体系中，吸附GOS后的材料也显示出比GOS/MIP和NIP更好的稳定性。

FE-SEM图像显示GOS/MIP（图6a、6b）为均匀的球形颗粒，粒径约为550 nm。TEM图像显示GOS/MIP具有核壳结构，Fe_3_O_4_核（粒径约410 nm）表面均匀包覆着厚度约为30 nm的SiO_2_层（图6c）和厚度约为40 nm的印迹层（图6d），这种特殊的形貌有利于传质优化和吸附位点暴露。能量色散光谱（图6e）显示，微球成功固定了Cu（Ⅱ）。元素面分布分析（图6f）显示Cu元素均匀分布在GOS/MIP上，这有利于印迹空腔的定向均匀，从而提升材料的选择性识别能力。磁滞曲线分析结果（图6g）表明，材料经层层包覆后仍能保持超顺磁性（47.78 emu/g），且在7 s内即可实现磁分离（图6g插图），极大降低材料的操作损耗，以及缩短制备和分离的时间。

**图6:**
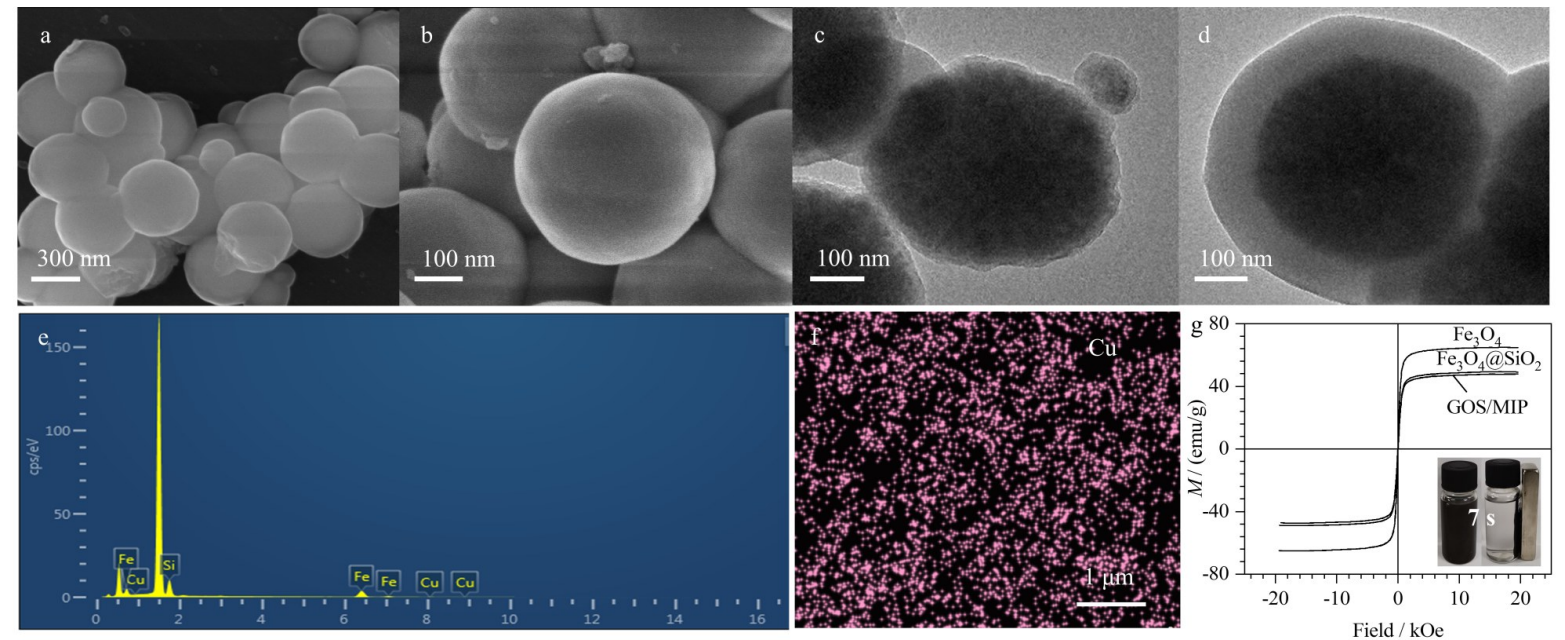
（a，b）GOS/MIP的扫描电镜图；（c）Fe_3_O_4_@SiO_2_和（d）GOS/MIP的透射电镜图；GOS/MIP的（e）能谱分析和（f）Cu元素分布；（g）GOS/MIP及载体的磁滞曲线（插图：磁分离示意图）

### 2.3 材料的结合性能

通过等温吸附、动力学吸附、选择性吸附和竞争性吸附实验考察了GOS/MIP的吸附性能和选择性识别能力。

热力学吸附实验（图7a）表明，材料的吸附量随着GOS溶液初始浓度的增大而增加，GOS/MIP的吸附能力显著高于NIP，归因于GOS/MIP中具有为GOS量身定制的印迹空腔，这些空腔在吸附过程中发挥重要功能。Scatchard方程分析显示，GOS/MIP为一条拟合曲线（图7b），归因于金属离子的引入使其结合位点定向均匀，同时印迹层位于载体表面使GOS能够很容易地到达特异性结合位点，所以强力的特异性吸附掩盖了弱的非特异性吸附，根据斜率和截距，可计算出GOS/MIP的表观最大吸附量（*Q*_max_）和解离常数（*K*_d_）值分别为74.01 mg/g和90.42 μg/mL。NIP只存在一种不可避免的非特异性吸附（图7c），其*Q*_max_和*K*_d_值分别是11.43 mg/g和48.31 μg/mL。通过拟合结果得到GOS/MIP的IF为6.48。上述结果表明，与NIP相比，GOS/MIP具有较强的特异性结合模板GOS的能力。

**图7:**
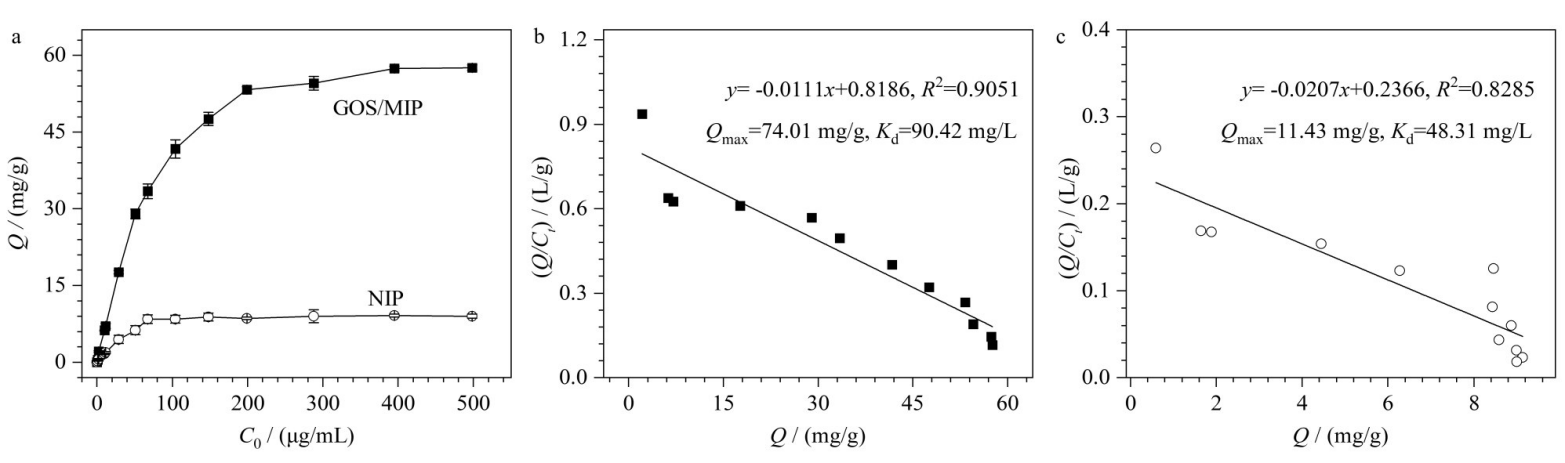
（a）GOS/MIP和NIP的等温吸附图； （b）GOS/MIP和（c）NIP的Scatchard拟合曲线（*n*=3）

动力学吸附曲线（图8a）显示，GOS/MIP在初始60 min内吸附量迅速达到平衡容量的70%，随后速率减缓并于约120 min时达到吸附平衡。这归因于在吸附初始阶段印迹空腔可高效捕获GOS，随空腔饱和吸附速率逐渐变慢。达到平衡时，GOS/MIP的吸附量（35.03 mg/g）显著高于NIP的吸附量（8.79 mg/g），这归功于所采用的配位键结合策略和表面印迹技术赋予了GOS/MIP高的特异性识别和结合GOS的能力，而NIP缺乏这种特异性结合位点。采用伪一级和伪二级模型拟合吸附动力学数据（图8b、8c）发现，两种材料都更符合伪二级模型，表明化学吸附可能是吸附过程中的速率控制步骤。

**图8:**
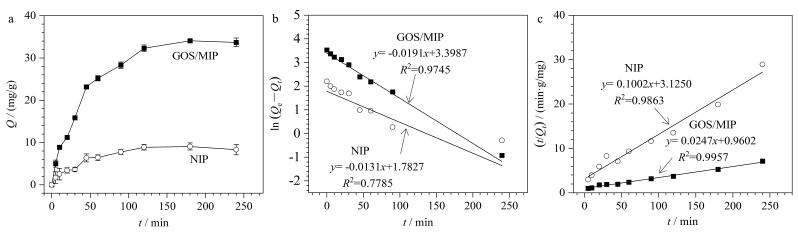
GOS/MIP和NIP的（a）动力学吸附图、（b）伪一级动力学拟合曲线和（c）伪二级动力学拟合曲线（*n*=3）

通过将GOS/MIP和NIP分别分散在GOS和其结构类似物（RUT、DOP、DOX、OTC、NFX、DHND、HNA，结构式详见图9）的溶液中进行选择性吸附特性研究，两种材料对各化合物的吸附量对比如图10a所示。结果显示，GOS/MIP对模板分子GOS的吸附量（约35.55 mg/g）显著高于对其他7种化合物的吸附量（均低于12.26 mg/g）。表明GOS/MIP对GOS具有较高的吸附选择性，与GOS分子尺寸及官能团精准匹配的印迹空腔保证了这种特性，而NIP没有表现出任何明显的优先吸附。

**图9:**
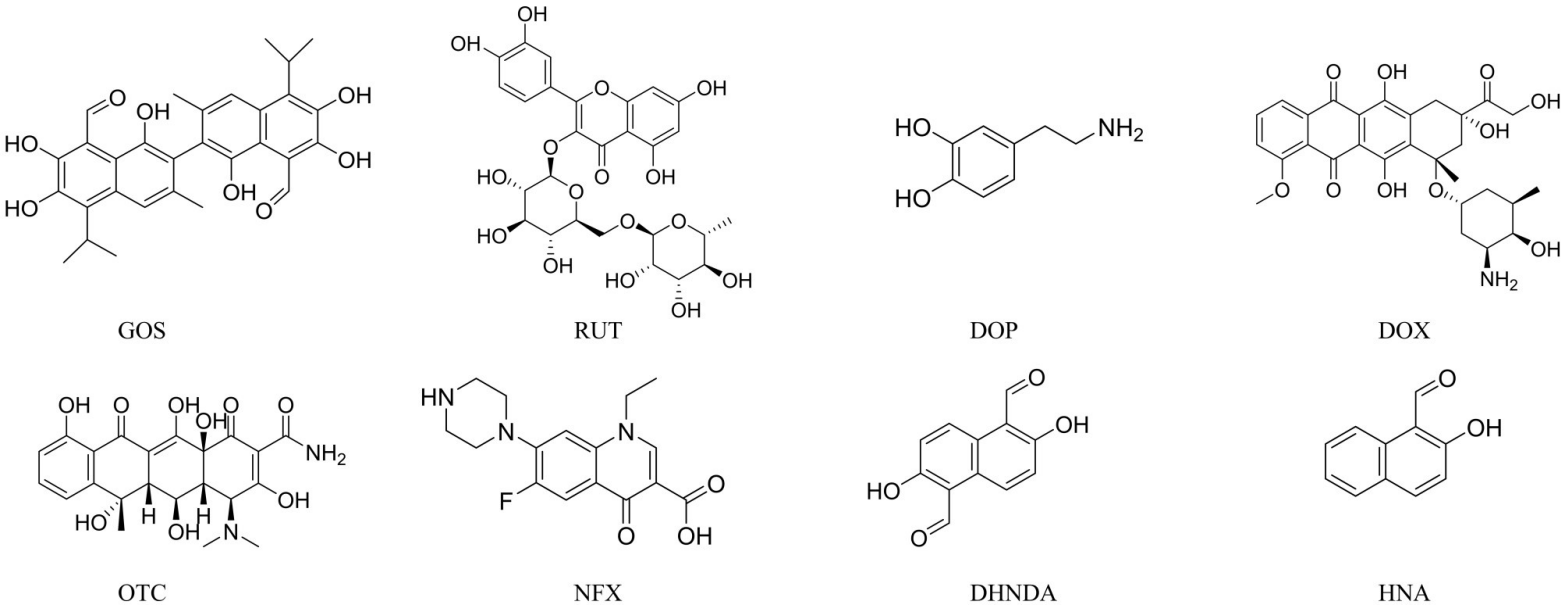
GOS和7种类似物的结构

**图10:**
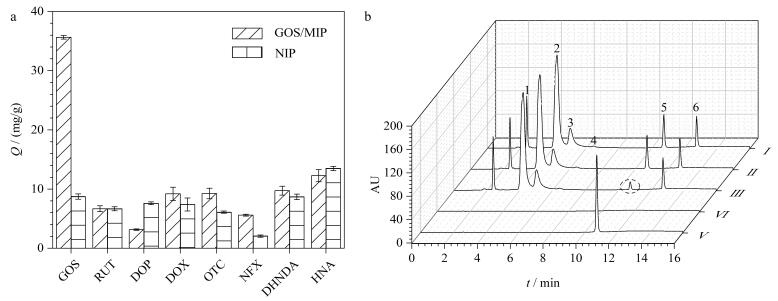
（a）GOS/MIP和NIP对GOS及类似物的吸附容量考察（*n*=3）； （b）竞争性吸附实验的色谱图

为了进一步评价GOS/MIP对GOS的特异性，将GOS/MIP和NIP分散在GOS、RUT、DOP、DOX、OTC和NFX的混合溶液中进行竞争性吸附实验。从图10b可以看出，经GOS/MIP处理后，溶液中仅GOS的浓度明显降低，洗脱液中仅出现一个与GOS对应的色谱峰，而NIP的洗脱液中几乎观察不到任何色谱峰。上述证据表明，GOS/MIP对GOS有特异性识别和选择性吸附作用，能从多种化合物的混合溶液中选择性地分离出GOS，克服复杂基质的影响。

### 2.4 pH适用范围测试

体系的pH值会影响吸附效率，导致吸附效果的不同。从图11a中可以看出，在pH 2~6的范围内，GOS/MIP对GOS的吸附量在pH=6时达最低值（27.83 mg/g），约为其最高值（35.55 mg/g）的80%，在此范围内能够保持较高效率的原因是GOS在这些pH条件下较为稳定，由此形成的配位复合物也比较稳定。而当pH值高于6.0时，配位复合物的稳定性被破坏，导致GOS无法被微球有效捕获。而NIP对GOS的吸附效率没有明显规律。

**图11:**
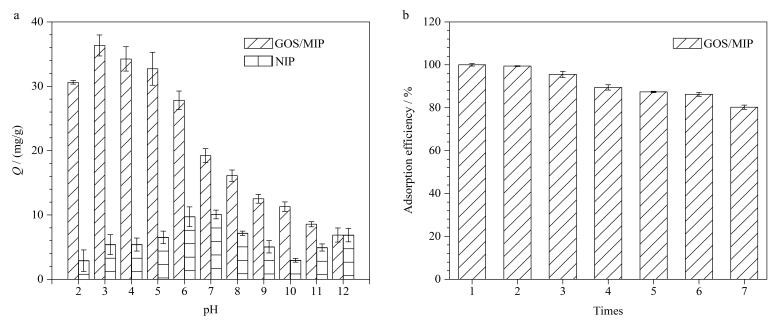
（a）GOS/MIP和NIP在不同pH条件下对GOS的吸附效率； （b）GOS/MIP的重复使用性（*n*=3）

### 2.5 重复使用性测试

吸附剂的可重复使用性是实际应用中的关键因素之一。如图11b所示，历经7次吸附-解吸-再生循环后，GOS/MIP的吸附容量仍保持在80%以上，表明即使反复吸附和洗脱导致部分结合位点被破坏，但再生的GOS/MIP仍然保持着良好的特异性识别GOS的能力，GOS/MIP具有良好的可重复使用性。

### 2.6 真实样本中GOS的分离

为了评估GOS/MIP的工业化应用潜能，将其用于从棉仁粗提物中分离获取GOS。首先建立了测定GOS的HPLC方法，标准曲线为*y*=2.088×10^4^ *x*‒6.158×10^4^（*R* ^2^=0.999，*x*：GOS的质量浓度，μg/mL，*y*：峰面积，mAU·min），线性范围为5~200 μg/mL，按照3倍和10倍信噪比（*S*/*N*）计算检出限（LOD）和定量限（LOQ），分别为0.024 μg/mL和0.079 μg/mL。为了评估GOS/MIP在实际样本中的适用性，将其用于从不同加标水平（0.08、0.24、0.80 μg/mL）的真实样本溶液（稀释至GOS质量浓度可忽略（0.009 8 μg/mL），以避免对加标回收的影响）中回收GOS。由表3可知，GOS/MIP对加标真实样本溶液中GOS的平均回收率为95.1%~98.7%，RSD为1.9%~2.4%，说明所建立的HPLC方法准确性良好，适用于GOS的分析。

**表3:** 棉仁的提取复溶溶液中GOS在3个水平下的加标回收率（*n*=3）

Spiked/ （μg/mL）	Detected/（μg/mL）	Recovery/%	RSD/%
0.08	0.0854	95.1	2.4
0.24	0.246	98.7	1.9
0.80	0.783	96.7	2.0

根据动力学吸附实验的结果，设置GOS/MIP与样本的孵育时间为120 min，可以满足规模工业化分离过程对时间的要求。如图12所示，GOS/MIP从棉仁粗提物中分离得到的GOS保留时间为7.48 min，与标准品（7.47 min）一致，样品中的GOS被成功回收。通过此方法测得棉仁中GOS的含量为0.50 mg/g。表4的结果表明，用50 mg GOS/MIP从10 g棉仁样品中分离得到3 mg GOS，回收率为77.0%~83.3%，纯度高于90%。上述结果证实了GOS/MIP是从棉仁中大规模分离得到高纯度GOS的有力工具。

**图12:**
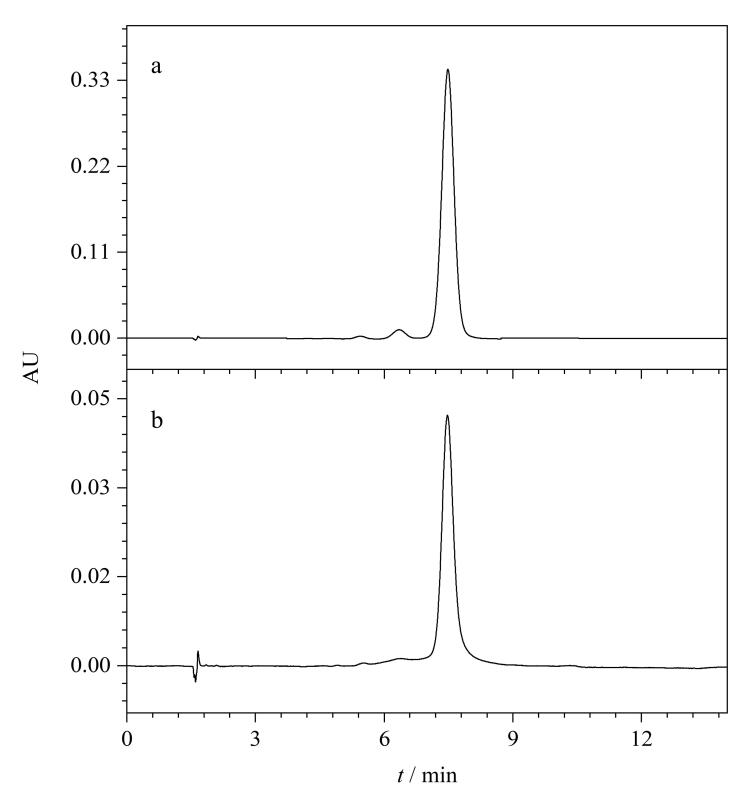
（a）GOS/MIP从棉仁样本中分离得到的GOS及（b）GOS标准品的色谱图

**表4:** 棉仁样本中GOS的分离实验（*n*=3）

*C*_（GOS）_/（μg/mL）	Measured/（μg/mL）	Recovery/%	RSD/%	Purity/%
92.1	21.2	77.0	0.08	94.1
97.7	22.9	76.6	2.2	92.1
107.6	18.0	83.3	2.6	91.0

### 2.7 与其他MIPs对比

相较于已报道的4种GOS的MIPs（表5），本文合成的材料通过表面印迹和金属介导作用提高了位点的可及性和分布均匀度，从而显著提高了IF值（6.48），虽然GOS/MIP的吸附容量不占优势，但是以特异性吸附为主，相较于其他几种MIP非特异性吸附最小（体现于IF最优），有利于从复杂真实样本中特异性、选择性地识别和分离GOS。配位键的高稳定性和高选择性略微牺牲传质速率，而更快实现平衡的两种MIPs略微牺牲了选择性。在今后的研究和材料设计中，可以适当减小磁核的粒径，增大比表面积，从而增加单位质量材料的有效印迹位点密度，提高吸附容量，同时，这也可使目标分子和印迹位点的接触更容易，从而加快传质速率、缩短平衡时间；此外，可以协同一些弱相互作用（如氢键），弥补单一强作用力的不足，以缩短平衡时间。

**表5:** 与其他关于GOS的MIPs报道的比较

Material	*Q*_max_/（mg/g）	Time/h	IF	Sample	Ref.
MIP-PMAA/SiO_2_	-	0.33	3.85	standard GOS	［^23^］
MIPs	632	12	1.3	-	［^25^］
MIPs	204	0.17	2.2	-	［^24^］
MIPs	120	0.67	2.86	-	［^26^］
GOS/MIP	71.32	2	6.48	cotton kernels	this work

IF： imprinting factor.

## 3 结论

本文通过表面印迹技术开发了一种铜介导的核壳结构磁性MIP（GOS/MIP），用于特异性识别和选择性分离GOS。Cu（Ⅱ）的介导有助于构建定向均匀的活性位点，同时，其引入的配位作用也有利于提高材料的识别能力和吸附性能。此外，基于配位键的可逆形成特性，该材料易于实现高效再生，使GOS快速进出空腔。通过模拟工业化生产过程，GOS/MIP可从植物样本中快速、高通量分离得到高纯度的GOS，为其规模化分离提供新的解决方案。
